# COVID-19-related sudden sensorineural hearing loss

**DOI:** 10.5339/qmj.2021.58

**Published:** 2021-10-25

**Authors:** Nada Khaleel Yaseen, Raid M. Al-Ani, Rasheed Ali Rashid

**Affiliations:** ^1^Department of Surgery/Otolaryngology, College of Medicine, Tikrit University, Tikrit, Iraq. E-mail: med.raed.alani2003@uoanbar.edu.iq; ^2^Department of Surgery/Otolaryngology, College of Medicine, University Of Anbar, Anbar, Iraq.

**Keywords:** COVID-19, sudden sensorineural hearing loss, tinnitus

## Abstract

Background: Sudden sensorineural hearing loss (SSNHL) can be a feature of COVID-19. It may present alone or with other symptoms of the disease. However, there is little written in the literature about its occurrence. We aimed to evaluate the socio-clinical characteristics and outcome of confirmed mild- to moderate COVID-19 cases with SSNHL in Tikrit city, Iraq.

Materials and Methods: This descriptive study was conducted at the Otolaryngology Department, Tikrit General Hospital, Tikrit city, Iraq. The period of the study was from December 1, 2020 to June 30, 2021.Mild and moderate COVID-19 subjects confirmed by real-time polymerase reaction were included in the study. Detailed demographic (age, gender, and smoking habit) and clinical characteristics (onset and duration of deafness, side, severity, associated ear, nose, and throat symptoms, and comorbidity) were recorded for every patient. Outcomes following the steroid treatment protocol were also registered.

Results: SSNHL was identified in 26 patients, of whom 20 (76.9%) were women, 20 (76.9%) were in the age group ≥ 30 years, and 21 (80.8%) were non-smokers. Around three-quarters of the subjects were identified within the first week of deafness occurrence. Bilateral (18/26) was more common than unilateral deafness (8/26); therefore, the total number of deaf ears was 44. Besides, bilateral symmetrical deafness (13/18) outnumbered the asymmetrical type (5/18). Around three-quarters were of moderate severity. The most common otological symptom was tinnitus (25/26). The most common nose and throat symptom was anosmia (6/26). The mean hearing threshold before and after treatment with oral steroids ± intratympanic steroids was 50.91 ± 11.777 dB and 40.24 ± 15.693, respectively. One patient with bilateral SSNHL was lost to follow-up; the remaining number of deaf ears was 42, and half of them were partially improved. The outcome of the treatment showed no statistically significant relation with the duration, side, and severity of SSNHL (*p*>0.05).

Conclusion: The majority of COVID-19-related SSNHL cases presented within one week of onset, with bilateral outnumbering unilateral cases. Tinnitus was the most common associated symptom. Treatment with steroids achieved partial improvement in half of the cases, and this outcome was not affected by the duration, side, and severity of deafness.

## Introduction

On March 11, 2020, the World Health Organization (WHO) declared COVID-19 a pandemic. This pandemic is caused by a single-stranded ribonucleic acid (RNA) virus. It is a highly contagious infection. By July 21, 2021, the WHO had registered 190,860,860 confirmed cases of COVID-19, including 4,101,414 deaths across the globe (https://covid19.who.int/). The most common presenting symptoms include fever, cough, dyspnea, fatigue, and olfactory and gustatory dysfunction. However, there is no uniform presentation of the disease in all infected individuals.^1^

Many prior investigations studied various otorhinolaryngological manifestations due to COVID-19. The most common manifestations include smell and taste abnormalities, dysphonia, sore throat, nasal obstruction, deafness, etc.^2,3^ The possible mechanisms of these manifestations are local viral inflammation, direct neural invasion, and Eustachian tube dysfunction. Therefore, it is not surprising to see one or more otorhinolaryngological symptoms in some COVID-19 patients.

Deafness can be caused by many viruses. These may cause congenital or acquired deafness of one or both ears; moreover, deafness may be sudden or gradual in onset. The possible mechanisms are a direct invasion of the internal ear, an inflammatory process that leads to its destruction and enhances the possibility of infection by bacteria or fungi. The usual type of deafness as a result of viral infection is sensorineural hearing loss (SNHL). However, other types of deafness (conductive or mixed) may be caused by some viruses. There is a persistent course of hearing loss in most cases, while spontaneous resolution may occur in some cases.^1,4–7^

In addition to viruses, there are many causes of SNHL, including labyrinthitis, trauma, noise-induced hearing loss, presbycusis, vestibular schwannoma, ototoxic drugs, metabolic and vascular insufficiency, and others.

Sudden SNHL (SSNHL) is one of the most frequent conditions seen in daily otology practice. However, the exact mechanism of the disease is not yet known. Viral infection is considered the most likely cause of this condition.^1^ Despite many scientific articles discussing SSNHL, there is little to be mentioned in the literature about SNHL due to COVID-19.^8^ A preliminary report from Thailand presented the case of an elderly woman with SSNHL due to SARS-CoV-2.^9^ Thereafter, many studies and case reports with cases of SSNHL as a consequence of COVID-19 were published.^10–13^ Non-specific symptoms of COVID-19, such as SSNHL can be associated with other features of COVID-19 or can be the only presenting symptom of the disease.^10^

Knowing the different symptoms of COVID-19 has a significant impact on reducing disease transmission and early treatment. These two goals have a positive effect on controlling and curing the disease. Furthermore, patients who presented with SSNHL alone might have COVID-19. This fact helps the otolaryngologist to send those patients for real-time polymerase reaction (PCR) test of naso- or oropharyngeal swabs in order to detect and isolate COVID-19-positive individuals.^10^ Hence, we conducted this study on confirmed mild and moderate COVID-19 patients with SSNHL. We aimed to assess the demographic, clinical, and treatment outcomes of SSNHL in the COVID-19-positive subjects.

## Materials And Methods

The retrospective study was conducted at the Otolaryngology Department, Tikrit General Hospital, Tikrit city, Iraq. This study was conducted over a period of 7 months (December 2020–June 2021). Out of 40,280 naso- or oropharyngeal swab PCR tests performed in the city of Tikrit during the study period, 4,850 new COVID-19 cases were identified. Confirmed mild and moderate COVID-19 cases complaining of SSNHL were enrolled in the current study. SSNHL is the deafness of at least 30 dB in at least three consecutive frequencies that has occurred within 3 days.^14^ All participants had normal hearing before acquiring the infection. Despite the retrospective nature of the current study, the study was approved by Ethical Approval Committee of the University of Anbar (reference number 90, 1-8-2021).

Patients with other causes of SNHL, age less than 18 years, other types of deafness (mixed or conductive), previous otological surgery, taking ototoxic drugs, pregnancy, age-related deafness, psychological disturbances, and those with incomplete data in their medical records were excluded from the study.

Detailed data including age, gender, smoking habit, onset, duration of COVID-19 and SSNHL, side of deafness (unilateral or bilateral), associated otorhinolaryngological symptoms, and comorbidities were recorded for every patient.

Physical examination including ear and tuning fork tests (Rinne, Weber, and absolute bone conduction tests), neurological, nose and throat, and systemic examinations were carried out for each subject.

Pure tone audiogram was performed for all participants using AD226 diagnostic audiometer 5500 Middelfart Denmark. The test was administered by a well-qualified technician in a well sound-proofed room with positive pressure aeration. The pure tone audiogram tests were carried out with the same COVID-19 protective measures (physical distancing of at least 1 meter, wearing personal protective equipment, and cleaning the surfaces with an antiseptic) to prevent the transmission of COVID-19 to the healthcare staff, other patients, and visitors. The average of the air and bone conduction curves at 500, 1000, 2000, and 4000 Hz were measured. The average air conductive curve at these frequencies was considered the threshold of the patient's hearing. The severity of deafness was classified into four grades according to the British Society of Audiology recommendation, where 20–40 dB is considered mild, 41–70 moderate, 71–95 severe, and >95 profound deafness.^15^ Other audiological tests [tympanogram using MAICO touch Tymp MI 24 Berlin, Germany and transient evoked otoacoustic emissions (TEOAEs) using MAICO DIAGNOSTIC GmbH Germany] were performed at Rahaf audiological center because these tests are not present in our hospital. These tests were carried out at the same protective measures of the COVID-19 in performing the pure tone audiogram.

All individuals were subjected to radiological investigations in the form of magnetic resonance imaging (MRI) with contrast of the ear and brain to show the state of the inner ear and exclude other pathologies.

The enrolled participants were treated with a loading dose of prednisolone (1 mg/kg/day with tapering of the dose over three weeks) as well as vitamin B-complex. For those patients who did not show an improvement, an intratympanic dexamethasone injection was administered under local anesthesia.

Thereafter, the subjects were re-examined for their threshold of hearing. Accordingly, the patients were classified into three groups: improved (threshold of hearing returned to normal), partially improved, and not improved.

SPSS (Statistical Package for the Social Sciences, IBM, Chicago, USA) version 25 was used for data analysis. The results were presented in simple tables or figures. The chi-square test was used for comparisons between categorical variables. A *p* value less than 0.05 was considered to indicate a statistically significant difference.

## Results

**Figure 1.**
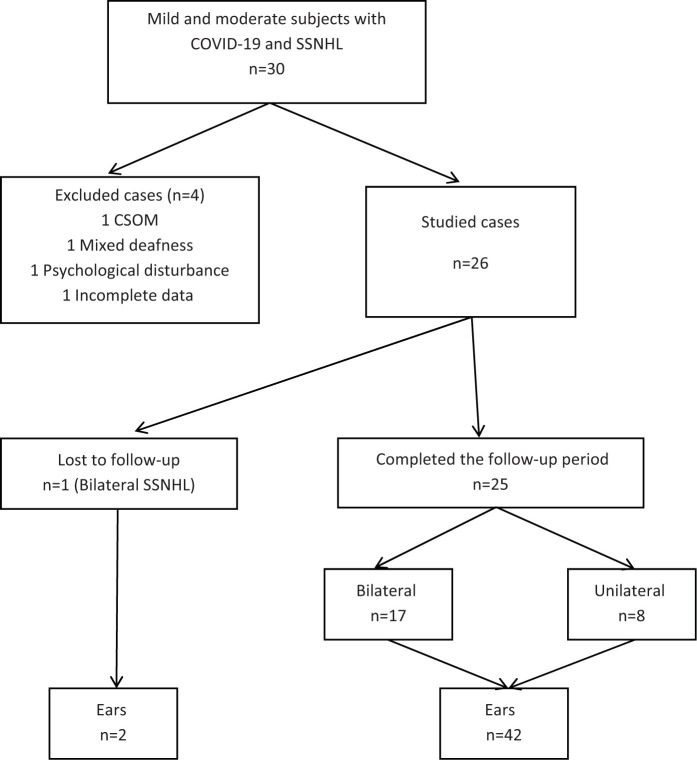
The studied subjects with SSNHL. CSOM = chronic suppurative otitis media and n = number.

Out of 30 mild to moderate COVID-19 patients with SSNHL, 26 fulfilled the inclusion criteria and were enrolled in the study (Figure 1).

The patients ranged in age from 21 to 66 years, and their mean age was 39.23 ± 11.884 years. Most of the cases were in the age group ≥ 30 years (n = 20, 76.9%), female (n = 20, 76.9%), and non-smokers (n = 21, 80.8%), as shown in Table 1.

The range of the duration of COVID-19 (duration that patients were COVID-19 positive) was 2–15 days, and the mean duration was 8.50 ± 3.972 days. The duration of SSNHL after COVID-19 was 1–9 days, and the mean duration was 4.15 ± 2.925 days. Most of the patients’ ears with SSNHL (n = 32, 72.7%) presented within the first 7 days from the onset of deafness. Bilateral was more common than unilateral deafness, and the symmetrical type (n = 13, 50%) was more common than the asymmetrical one (n = 5, 19.3%) (Table 2). Therefore, the total number of ears involved in the study was 44 (Figure 1). Moderate severity of SSNHL was the commonest (n = 32, 72.7%), while the least severe form was the least common (n = 1, 2.3%). The majority of the patients had tinnitus (n = 25, 96.2%). Vertigo was present in 11 (42.3%) cases. Anosmia, dysphonia, and ageusia were present in 6 (23.1%), 2 (7.7%), and 1 (3.8%) case, respectively. The majority of patients did not have hypertension and diabetes mellitus (n = 25, 96.2%) (Table 2).

**Figure 2.**
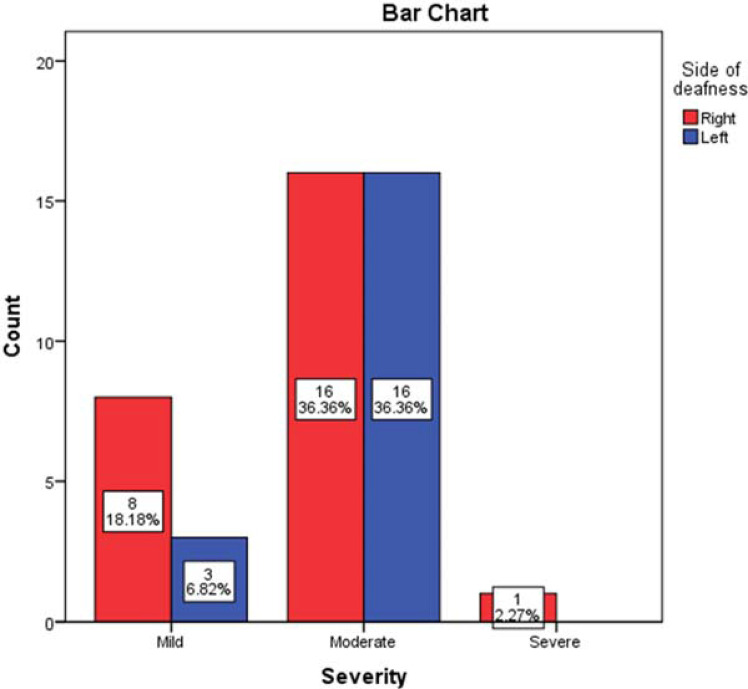
Relationship between the affected side and severity of SSNHL in the 44 ears (*p* = 0.286).

The mean of the average hearing loss before starting treatment was 50.91 ± 11.777 dB (total range 25–80). Following treatment, the total range of the average hearing loss was 10–80 dB (mean = 40.24 ± 15.693). There were 25 (56.8%) diseased ears on the right side. Moderate severity was the most common on both sides (n = 16, 36.36% for each side). There was no statistically significant relation between the affected side and the severity of SSNHL (*p* = 0.286) (Figure 2). All patients showed a type A tympanogram in both ears, reduced amplitude on TEOAE, and no abnormalities on MRI with contrast of the labyrinth and brain examination.

**Figure 3.**
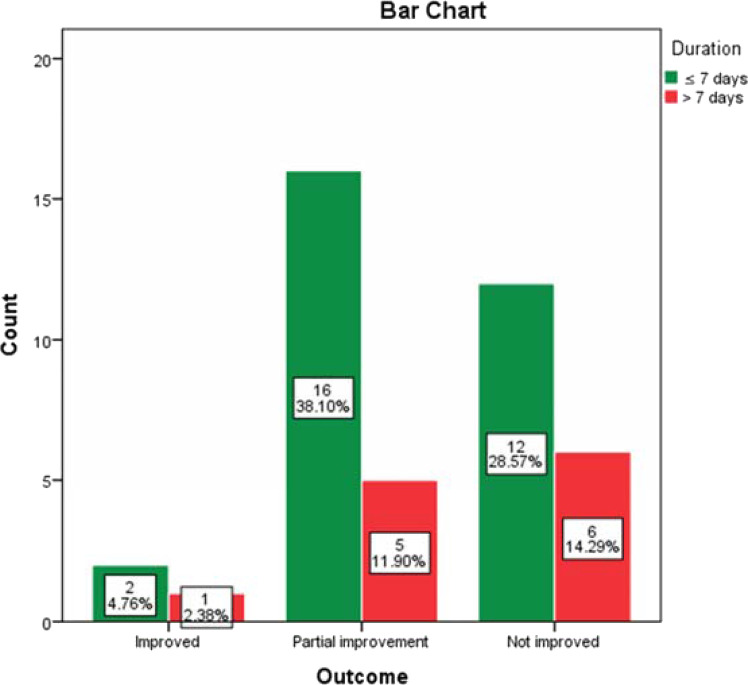
The relationship between the duration and outcome of the SSNHL in the 42 ears (*p* = 0.792). One patient with bilateral SSNHL lost to follow-up.

**Figure 4.**
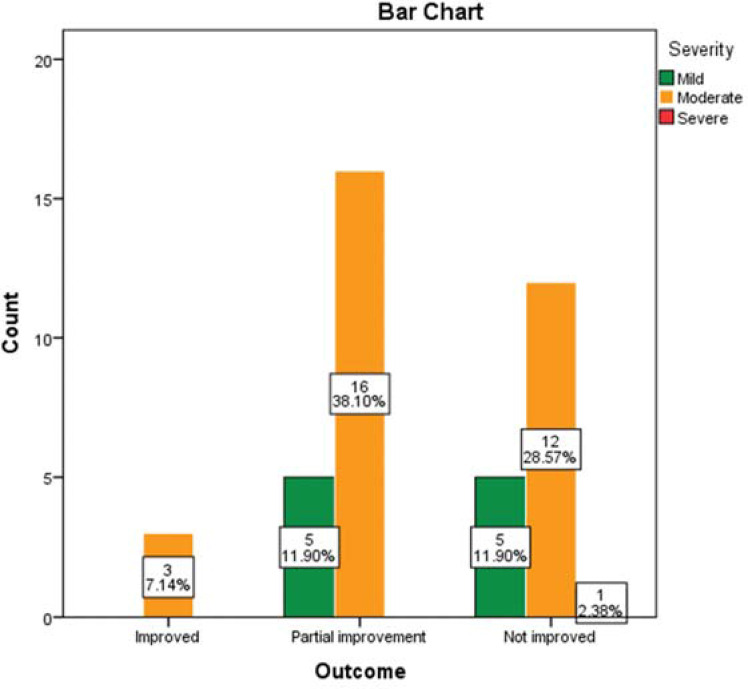
The relationship between the severity and outcome of the SSNHL in the 42 ears (*p* = 0.629). One patient with bilateral SSNHL lost to follow-up.

**Figure 5.**
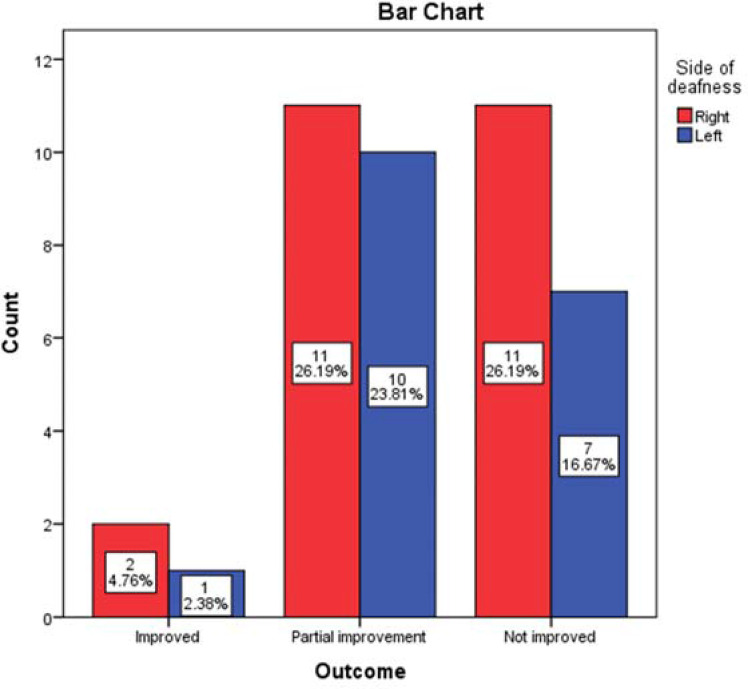
The relationship between the side and outcome of the SSNHL in the 42 ears (*p* = 0.810). One patient with bilateral SSNHL lost to follow-up.

The majority of patients were partially improved (21, 50%) on oral prednisolone with or without intratympanic injection of dexamethasone. However, the outcome of the patients was not significantly related to the affected side, duration, and severity of deafness (*p*>0.05) (Figures 3, 4, and 5).

## Discussion

Otorhinolaryngological manifestations are well-known features of COVID-19 with different prevalence rates. Chemosensory dysfunctions (smell and taste abnormalities) are the most common feature of the disease. Other features such as nasal obstruction, sore throat, dysphonia, and deafness are less likely to be the result of COVID-19.^16^ SSNHL is reported in the literature, but mainly as case reports or small case series. To the best of our knowledge, this study is the largest case series so far despite its small sample size. The main outcomes of the current study were that SSNHL occurred more often in the age group ≥ 30 years old, women, and non-smokers. Bilateral was more prevalent than unilateral deafness, and the symmetrical type occurred more often than the asymmetrical form. Half of the cases were partially improved following the regime (oral steroids with or without intratympanic steroids within two weeks from the onset of deafness) recommended by the American Academy of Otolaryngology-Head and Neck Surgery clinical practice guidelines.^17^

SSNHL commonly involves one ear.^17^ Although rare, bilateral deafness raises the suspicion of vascular, infection, inflammation, ototoxic drugs, trauma, tumors, and autoimmune diseases.^14^ The majority of the reported cases of SSNHL following COVID-19 were unilateral.^8,10,18,19^ By contrast, our study revealed that the majority of the patients had bilateral SSNHL (13/26 symmetrical and 5/26 asymmetrical). Bilateral SSNHL could be due to the deleterious effect of SARS-CoV-2 on the outer hair cells of the cochlea.^12^ Chern et al. reported an 18-year-old COVID-19-positive female patient with bilateral asymmetrical SSNHL with intratympanic bleeding confirmed by MRI,^13^ and Degen et al. revealed a 60-year-old COVID-19-positive man who presented with bilateral SSNHL and loud tinnitus.^11^

Cohen et al., in a review study regarding the viral causes of deafness,^20^ found that the incidence of SNHL in the cytomegalovirus (6%–23% in asymptomatic and 22%–65% in symptomatic individuals), HIV 27.5%–33.5%, herpes simplex virus 33%, rubella 12%–19%, measles 0.1%–3.4%, and mumps 0.005%–4%. Moreover, Swain et al. from India first reported that the prevalence of the SSNHL was 2.45% of the confirmed COVID-19 cases (16/652). However, the prevalence of the SSNHL was much lower than the prevalence of other otorhinolaryngological manifestations due to COVID-19 like smell, taste, and dysphonia.^16,21^ As reported in the literature, there are three main mechanisms of SSNHL due to viral infections, namely neuritis due to viral infection of the cochlear nerves, perilymphatic tissue involvement by a viral infection, and stress response due to cross-reactions of the antigens of the labyrinth.^22^ Furthermore, viruses might reach the internal ear and cause deafness, either by direct invasion or indirectly through cerebrospinal fluid as reported by researchers in the animal models.^23,24^ Despite the abovementioned mechanisms, many autopsy studies on the temporal bones of subjects who suffered from idiopathic SSNHL failed to find abnormalities such as cytopathogenicity, viral particles, specific viral antigens, and viral nucleic acids.^4^ Moreover, similar histopathological results were detected in the temporal bones of patients with idiopathic SSNHL and those with deafness due to certain viruses like rubella, herpes zoster, measles, and mumps.^25^ Our radiological findings revealed no abnormalities detected on the MRI images in any patients. Nevertheless, Swain et al. showed that MRI with contrast revealed inflammatory changes of the cochlea in 62.5% of the studied cases.^19^ Moreover, reported cases with SSNHL due to COVID-19 showed various findings such as bilateral intralabyrinthine hemorrhage^13^ and enhancement of the right and normal hypointense of the left cochlea in a patient with bilateral SSNHL.^11^ We can conclude that in the majority of SSNHL cases due to COVID-19, there is no identified pathology in the cochlea.

The mean age in the study by Swain et al. was 48.42 years (age range 38–72 years), and 56.25% of the patients older than 50 years, which was higher than in our study (age range 21–66 years, mean 39.23 ± 11.884 years old, and 70% of cases older than 30 years). The difference in the age distribution between the two studies may be attributed to the difference in the inclusion criteria, mild to moderate cases in the present study vs. hospitalized cases in the study by Swain et al.^19^ As mentioned above, SSNHL due to COVID-19 can affect patients of any age. Besides, there was a gender distribution difference between the study of Swain et al (68.75% males) and the current one (76.9% females). This discrepancy may be due to geographical and ethnic differences.

SSNHL due to COVID-19 involves various degrees of severity from mild to total deafness, according to the cases reported in the literature.^8,10,11,13,26^ Approximately 25% of our cases were mild SSNHL. The mild severity could be the cause of a delay in the diagnosis of SSNHL, particularly if it is unilateral. Therefore, every patient presenting with SSNHL should be sent for an oropharyngeal or nasopharyngeal swab PCR test to confirm or exclude COVID-19 as the cause of deafness.

A study by Umesawa et al. reported that the prevalence of current smokers of both sexes was significantly higher in patients with idiopathic SSNHL than in the control group. Moreover, the smoking habit among males had a positive association with the severity of deafness.^27^ In the current study, non-smokers outnumbered (21/26) smokers (5/26); this may be attributed to the fact that the highest proportion of our cases were women (women are less likely to smoke than men).^28^ The study did not investigate the association between smoking and SSNHL, because it was out of the scope of our study. Moreover, prior studies failed to find a significant association between smoking and other otolaryngological symptoms due to COVID-19, such as smell, taste, and dysphonia,^21,16^ despite the deleterious effect of cigarette smoking on the olfactory and upper respiratory epithelium.^29^

The nose and throat symptoms (anosmia, dysphonia, and ageusia) associated with COVID-19-related SSNHL revealed by our study might explain the neuroinvasive nature of the virus.

Vertigo is considered as one of the poor prognostic factors in patients with idiopathic SSNHL. Vertigo has a deleterious effect on the initial hearing threshold. However, it has no direct effect on the treatment outcome of the disease.^30^ The high prevalence of vertigo in our study (42.3%) might be considered as one of the poor prognostic factors of COVID-19-related SSNHL. Nevertheless, the current study did not report a significant association between the treatment outcome with steroids and the side, duration, and severity of SSNHL due to COVID-19.

The occurrence of SSNHL in COVID-19 patients, either as a sole manifestation or associated with other features of the disease, has two important clinical implications.^10,19^ The first is home or hospital quarantine (depending on the condition of the patient) of any subject with SSNHL in the era of the COVID-19. The second is that all the necessary physical examinations and investigations should be undertaken with full protective measures against SARS-CoV-2 to block the transmission of the infection from infected to healthy individuals.

## Conclusion

This study showed that the majority of COVID-19-related SSNHL cases were women, in the age group ≥ 30 years, and non-current smokers. Patients presented within one week of acquiring the infection in most cases, with bilateral being more frequent than unilateral cases. Tinnitus was the most common associated otorhinolaryngological manifestation. Treatment with oral prednisolone with or without intratympanic dexamethasone injection achieved partial improvement in 50% of individuals. The high frequency of vertigo might be a poor prognostic factor. There was no significant association between the outcome and the duration, side, and severity of COVID-19-related SSNHL.

## Figures and Tables

**Table 1:** Demographic characteristics of the 26 COVID-19 patients with SSNHL.

Variable	Number (n–26)	Percentage

**Age groups (years)**		

< 30	6	23.1%

≥ 30	20	76.9%

**Gender**		

** Male**	6	23.1%

** Female**	20	76.9%

**Smoking habit**		

Yes	5	19.2%

No	21	80.8%


**Table 2:** Clinical characteristics of the 26 COVID-19 patients with 44 ears with SSNHL.

Variable	Number	Percentage

**Duration of SSNHL (n = 44 ears)**		

≤ 7 days	32	72.7%

>7 days	12	27.3%

**Side of deafness (n = 26 patients)**		

Right	7	26.9%

Left	1	3.8%

Bilateral symmetrical	13	50.0%

Bilateral asymmetrical	5	19.3%

**Severity (n = 44 ears)**		

Mild	11	25.0%

Moderate	32	72.7%

Severe	1	2.3%

Profound	0	0

**Tinnitus (n = 26 patients)**		

Yes	25	96.2%

No	1	3.8%

**Vertigo (n = 26 patients)**		

Yes	11	42.3%

No	15	57.7%

**Anosmia (n = 26 patients)**		

Yes	6	23.1%

No	20	76.9%

**Ageusia (n = 26 patients)**		

Yes	1	3.8%

No	25	96.2%

**Dysphonia (n = 26 patients)**		

Yes	2	7.7%

No	24	92.3%

**Hypertension (n = 26 patients)**		

Yes	1	3.8%

No	25	96.2%

**Diabetes mellitus (n = 26 patients)**		

Yes	1	3.8%

No	25	96.2%

